# Advancing the State of the Fog Computing to Enable 5G Network Technologies

**DOI:** 10.3390/s20061754

**Published:** 2020-03-21

**Authors:** Yahui Meng, Muhammad Ali Naeem, Alaa Omran Almagrabi, Rashid Ali, Hyung Seok Kim

**Affiliations:** 1 School of Science, Guangdong University of Petrochemical Technology, Maoming 525000, China; mengyahui@gdupt.edu.cn (Y.M.); malinaeem7@gmail.com (M.A.N.); 2 Department of Information Systems, Faculty of Computing and Information Technology (FCIT), King Abdulaziz University, Jeddah 21589, Saudi Arabia; aalmagrabi3@kau.edu.sa; 3 School of Intelligent Mechatronics Engineering, Sejong University, Seoul 05006, Korea; rashidali@sejong.ac.kr; 4 Department of Information and Communication Engineering, Sejong University, Seoul 05006, Korea

**Keywords:** Fog computing, 5G networks, computing paradigm, IoT

## Abstract

Fog Computing (FC) is promising to Internet architecture for the emerging of modern technological approaches such as Fifth Generation (5G) networks and the Internet of Things (IoT). These are the advanced technologies that enable Internet architecture to enhance the data dissemination services based on numerous sensors generating continuous sensory information. It is tough for the current Internet architecture to meet up with the growing demands of the users for such a massive amount of information. Therefore, it needs to adopt modern technologies for efficient data dissemination services across the Internet. Thus, the FC and 5G are updating the data transmission using new technological approaches that are intelligently processing data to provide enhanced communications. This study proposes necessary measures to boost the growth of FC to 5G network usage. It is done by taking an extensive review of how 5G operates as well as studying its taxonomy, the idea of IoT, reviewed projects on IoT applicability, comparison of computing technologies, and the importance of FC. Moreover, it elaborates dynamic issues of computing network technologies, and information is provided on how to remedy these for future recommendations in the field of research and computing network technologies. This paper heavily focuses on the applications of FC as an enabler to the 5G network by identifying the necessary services and network-oriented features that are needed to be used in the place for an improved future enterprise network technology.

## 1. Introduction

In recent times, the Internet has facilitated communication in our daily lives with the help of computers, laptops, and smart devices. The increased usage of smart devices envisages being around 10 to 12 billion by 2021 [1]. However, it is impossible for the present computer networking technologies to support the overtly growing demands and usage of these devices. Thus, existing network technologies need to adapt the current networking technologies to meet up with the stringent needs of future use cases. An improved computing model is required to enable smart devices to use the upcoming 5th Generation (5G) network.

The 5G is a plausible computer networking technology for the future that expects to power ultra-fast smart devices. It is also aimed at reducing latency to sub-milliseconds, in return reducing infrastructure bottlenecks. 5G networks aim to tackle these challenges by introducing very high carrier frequencies with cutting-edge technological functionalities like fogging, i.e., Fog Computing (FC) and Edge Computing (EC), and Device-to-device (D2D) communication [2]. It requires middle layer intelligence to achieve these cutting-edge technological functionalities. The middle layer intelligence will receive data input from the remote distributed Internet of Things (IoT) network that is backed-up with the data layer. This IoT network can be processed anywhere at the edge nodes of the network. Moreover, 5G wireless network envisages bringing opportunities in the domain of wireless communication technology like never seen before. It is assumed to achieve the aims such as increase the growth of system capacity, long-lasting with efficient energy retention capabilities, curtail more latency, and extra throughput compared with existing versions of wireless networks, such as 4G/3G/2G [3].

With the constricted resources of mobile terminals, 5G technology would provide challenging services and applications, especially for FC [4]. 5G promises to overcome faults of resource constraint present in wireless computing network by offering enhanced resource-studded with an array of dazzling services [5]. This heavily focuses on premier quality wireless communication with an offer of superior spectral efficiency. As demonstrated in [6], a propped-up Fog Radio Access Network (F-RAN) would merge FC into a diversified cloud radio access network. Moreover, resolving the current challenges of the centralized base-band unit pool, it also finds a solution for the challenges related to Cloud Radio Access Network (C-RAN) with the constricted front haul. It does this by making the actual-time collaboration available between flexible, cooperative radio resource management and radio signal processing at the devices that are at the edge.

FC is one of the modern technologies which have gained rapid growth in a brief period. FC enables the effective processing of data retrieved from smart devices. It brings computing paradigm from the cloud to the edge of the network with computing capabilities. However, massive data generation from numerous sensors and actuators in the Internet of Things (IoT) has first networks to a bottleneck [7,8]. Thus, the need to improve computing server architecture to handle the continuous growing demands of smart devices is pertinent. Therefore, in this study, some cases of fog computing on 5G networks are discussed. Currently, using FC technology in 5G systems has much vision, and the same goes for the 5G network itself. Therefore, to actualize this vision, a few of the research areas needed to be addressed. This study provides comprehensive knowledge about FC technologies that are used to serve as an enabling computing technology to the 5G network [3,6]. The study elaborates on dynamic issues in the field of computing network technologies, and information is provided on how to remedy these for future recommendations in the area of research and computing network technologies. In addition, this study will contribute to providing the knowledge that helps to emerge the modern technologies to deliver efficient data dissemination services. For example, the concept of Network Function Virtualization (NFV) in heterogeneous networks using FC as an enabler to 5G is not clear in present times since there is a need for improvement in implementing NFV for mobile communication networks as explored in earlier studies [9,10,11]. Moreover, a large amount of energy is required for the processing of more substantial applications [12]. Minimizing energy consumption is key for commercializing FC technology in 5G systems. Another challenge is the pricing policies because different parties require different prices of resources for the use of FC services [13]. Thus, finely granulated accounting management and price policy are needed in resource management of the FC and 5G ecosystem. Finally, the issue of visibility of data to the third party by 5G networks makes privacy and security a pertinent point in FC architectures [14]. These issues and challenges can be addressed using FC in 5G networks. It is evident that both technologies, i.e., FC and 5G, are compatible with each other. However, combining both concepts is envisaged to be the future of Internet services. The present study aims to find a better solution and a more sustainable future for humanity.

This paper is structured as; Section 1 gives a brief introduction about the requirements of present study. Section 2 provides a brief review of related study. Section 3 presents a review related to 5G, importance of IoT, applicability (on-going projects) and a brief SWOT analysis on the existing computing network paradigms. Section 4 explains why Fog is envisaged to be a better enabler to 5G networks by giving examples of its existing use cases. Section 5 gives an overview of FC. In Section 6, several use cases of FC are explained. Section 7 elaborates how the convergence of Fog and 5G network can be actualized. In Section 8, some of the future research directions are described. At the end, in Section 9 the study is concluded.

## 2. Related Research

Several survey papers have recently been published to provide the functionalities of FC. Moreover, these surveys provide information about FC technologies. Moreover, these surveys have broad and limited scope to treat FC partly. However, the present paper offers comprehensive knowledge about FC technology and provides multiple solutions to meet the future Internet and end-user requirements. Moreover, this study provides a complete survey of emerging technologies such as 5G and the Internet of Things. Also, this study tells how FC with 5G is benefitted for the content dissemination across the edges of the Internet and provides fast communication services. This survey focuses on the critical analysis of the IP-based Internet architectures and its emerging technologies.

Table 1 presents a few of the related studies by identifying details and areas of discourse. An overview of how to resolve the challenges related to computer network technology, Fog architecture, quality of services, and experience were proposed in this study. For existing studies, this study provides a persuasive explanation of how to advance FC as an enabler of 5G networks.

## 3. 5th Generation (5G) Networks

The emergence of different technologies for transmitting data into a localized small network via Wi-Fi, CBRS, and 5G has helped in the advancement of Information Technology in recent times. However, these technologies have their benefits and challenges in the wireless ecosystem. The roadmap to 5G is basically to secure better Internet usage [7]. It is significant to know that this technology was introduced to the networking ecosystem as far back as in the early 1980s’. It evolved from the First Generation (1G) to the on-going deployment of the Fifth Generation (5G). The 1G supports the voice calls with speed up to 2 kbps. The 2nd-Generation (2G) network founded in the early 1990s, which supports voice calls, SMS text messaging with speed up to 64 kbps. The 3rd Generation (3G) network communication technology founded in the 2000s, and it is still active until the present era. 3G network supports the voice calls, SMS text messaging with a speed up to 64 kbps as seen in [1,15,17]. The 4th Generation (4G) network communication technology developed in 2010, and it is also active until the present in many of the developing as well as developed countries. 4G functionality supports voice calls, SMS text messaging, videos, mobile Internet, Bluetooth connectivity with speed up to 1 Gbps. The 5G computing network is undergoing deployment and yet on the way to deploy. It is anticipated to support voice calls, SMS text messaging, videos, mobile Internet, Bluetooth connectivity with a speed up to 10–100 Gbps as mentioned in [1,2,17,18,19]. Researchers consider 5G as a better wireless broadband network. It is standardized by ITU’s Third Generation Partnership Project (3GPP). However, it is the next generation of wireless technology that will be deployed commercially on a large-scale, which is expected to be rolled out within the time-frame of 2019–2020 [17]. 5G is an advancement of other generation wireless broadband networks. The immediate applications of 5G will be to enhance fixed broadband services and mobile broadband speed by offering a better solution for network enterprise with improved functionalities. These features can be classed into phases such as:

1. Enhanced Mobile Broadband (eMBB)
2. Ultra-Reliable Low-Latency Communications (URLLC) or ultra-Machine Type Communications (MTC)
3. Massive Machine Type Communications (mMTC).

A greater data-bandwidth is provided at the initial phase of deploying 5G networks that are heavily focused on the eMBB. eMBB complements a moderate latency improvement on both 5G new radio (NR) and 4G long term evolution (LTE). It has helped in developing mobile multimedia broadband, such as AR/VR media applications, 360° streaming videos, and others. mMTC has developed as a part of 3GPP with a Low Power Wide Area (LPWA) technologies. They are predicted or expected to meet most of the 5G requirements. In contrast, others require more bandwidth with ultra-reliable low-latency communication (URLLC) requirements that will be included in 5G Core deployment for full end-to-end latency reduction [14]. Computing networks should meet the requirements to support 5G networks to enhance mobility, delay critical communication applications, security, and data protection. However, to give a detailed review of computational technologies, other computing paradigms needed to be reviewed. Primarily, it is important to know why devices are needed to be connected by allowing all potential objects to interact with each other in the IoT environment.

### 3.1. IoT-Based Issues Related to 5G

IoT covers a wide area of domains as well as fields of study. The dynamicity of IoT faces various setbacks in relation to devices resource constraint and many more [20,21]. Considering the huge amount of data that needs to be continuously collected in other to guarantee an effective system that makes the actual decision-making process, technologies like Cloud Computing (CC), EC and FC provide solutions to the limitations of IoT devices [22]. These computing paradigms (CC, EC, and FC) perform data gathering tasks from the user device to enable accurate decision-making as well as producing the outcome or results to user devices [23].

**Figure 1:**
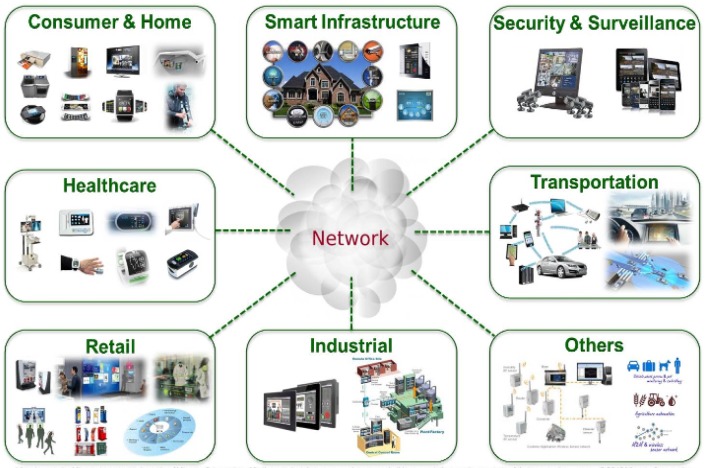
Consumer IoT applications.

This idea gives birth to what is known as the consumer IoT. Consumer IoT refers to the increasing number of personal users of smart devices that are connected via the use of the Internet to share and collect data for personal use [24]. Figure 1 illustrates the approaches of the consumer IoT environment. However, the management layer interposition between computing paradigms and user devices may be a barrier to meet data transmission quality of services (QoS) requirements. Computing paradigms like CC and EC, most of the time, fail at supporting the geo-distribution and mobility of user devices, as mentioned in [8,22,23].

To support the billions of devices, it expected that the Internet would include critical problems related to resource management in 5G networks. It will become more serious when these devices operate within wireless environments. Many IoT devices are needed to be connected using wireless communication technologies, where communication becomes inconvenient through wired links such as communications in smart buildings, houses, and factories. Soon, it is expected that the IoT will be dependent on the wireless/mobile Internet. In these situations, 5G is the most significant platform that can overcome the expected issues related to wireless communication of billions of IoT devices [25].

Security is one of the critical issues for the IoT-based environment. In the IoT environment, all the connected things are controlled using sensors such as the temperature sensor attached in a smart car. It looks like a straightforward environment, but if we are dealing with some severe problems such as in defense, security needs to be addressed significantly. For example, sensors that measure the temperature might not need high security, such as required in the healthcare environment. Therefore, a high level of protection needs high complexity, and presently, it is necessary to address the IoT environments. Thus, 5G is considered to be the future platform to enhance the IoT-based environments. The reason is that the data traffic is continuously increasing, which increases the interference due to immense signaling generated from IoT devices [26]. Therefore, it will be difficult for the IoT environment to handle these issues efficiently. On the other hand, 5G is a new cellular technological approach that can solve such problems with high efficiency.

Scalability is also a critical problem in an IoT-based environment, and it is needed to be addressed. It refers to the capability to present complex applications and devices in the IoT environment without relating to the quality of services [27]. Thus, it requires a standard system to manage and maintain control of the massive number of connected devices. Hence, 5G provides a better way to handle these issues in an efficient manner [28].

Network management is used to manage network resources such as service and devices. According to the IoT concept, in which many devices and their diverse categories of connections have significant management needs such as Configuration, Fault, Accounting, security, and Performance. Hence, in a conventional network, resource management such as remote control, maintenance, and monitoring are significant in the connection of heterogeneous devices in an IoT-based environment. The reason is that it is challenging to manage such a massive number of different devices because these devices must cater, control, and succeed for distinctive features of the IoT environment. Therefore, it requires a new environment to manage these devices efficiently [29].

Interoperability is one of the most significant problems within in heterogeneous network environment in which everything needs to connect everywhere because these devices need end-to-end interoperability among the diverse network technologies. The IoT-based heterogeneous devices must connect using some communication technology to communicate and disseminate the information. In this situation, 5G is a beneficial approach to achieve high interoperability for IoT-based environments [30].

Coverage and mobility are the essential concerns that until remain as challenging for the IoT-based environment to deploy the communication services. It is a crucial premise for the IoT environment to have an efficient connection during the devices’ movement. Consequently, IoT-based mobile devices have been facing interruption while moving from one gateway to another. Therefore, an enhanced mobility management approach is required to control and manage the vast number of connected devices in an IoT-based environment. As a result, the current 5G cellular network is a promising approach to provide efficient services for mobile IoT-based environments [31].

### 3.2. Research Projects on IoT

Recently, researchers have carried out projects to manage and organize the challenges of smart cities. SmartSantander [32,33] is a project that is used to help the architectural model of IoT and the inherent issues of provision of resources in smart cities. The framework for this project provides an appropriate platform for large-scale testing and evaluation of IoT. Its use cases are deployed in many urban areas. Another project is known as CityPulse project [8] that is focused on working the data analytical framework for smart cities. It integrates data aggregation with powerful analytical tools as well as modules for event detection and quality assessment algorithms. It aims to develop and support customized smart city applications. Another application is currently working on the resilient IoT SusCity project [34]. This project is heavily focused on data collection from diverse sources to develop an intelligent management solution for appropriate decision-making to help the government and citizens. Another project known as VITAL federates heterogeneous IoT platforms through a cloud-based semantics environment focusing on smart cities [35]. A large-scale network composed of Fog/cloud platforms and SDN technologies is presented in a study reviewed by [8]. The objective of this study was to minimize the amount of redundant data as well as reduce the response time in accessing data services. Thus, the research focused only on the configuration of services in Big Data. The aim of our research is not to channel only towards data analytics but also to decision-making for autonomous management of smart city applications in different ways. Their work is based on simulation, while our research is focused on actualizing the deployment of FC with a 5G network for IoT use cases. In an article concerning how 5G can be used in smart cities [8], an approach was proposed using M2M communication with cognitive 5G networks. This study developed an approach that involved a ‘decentralized multiple gateway assignment protocols based on multi-channel Carrier Sense Multiple Access’ (CSMA). The objective of this study is to increase the system throughput and reduces the energy consumption by minimizing payloads of message headers, and a low overhead protocol was also proposed. The simulation was carried out to evaluate the effectiveness of the proposed approach in terms of network lifetime and energy consumption. This study has produced enhanced results that will be significant for future directions towards further studies in the same field of research.

The European Commission alongside public–private partners, manufacturing industries, Internet service providers (ISP), and researchers is established the 5G infrastructure Public–Private Partnership (5G-PPP) to improve the research under 5G technologies in European countries as well as develop a global consensus on its networks [22]. This project aims to proffer solutions and standards to the ubiquitous next-generation communication infrastructures in the future. With regards to management and compositions, 5G-PPP will be able to highlight issues using developed projects to address varying network concerns as given below:

- The delivering of 5G virtualized networks for agile service development as well as employing a virtualization software known as (SONATA) [16]. SONATA optimizes software network by making it flexible for programmability for development and deployment.
- The development and validation of novel control framework for mobile networks in the next generations using a coordinated control and spectrum management for 5G heterogeneous RAN (COHERENT) project [36]. The importance of the COHERENT is to manage the underlying heterogeneous mobile networks.
- Another project called the ‘Self-organized Network Management in Virtualized and Software Defined Networks’ (SELFNET) [4]. This project was developed for self-organizing capabilities using an implemented autonomous network management framework. Its features include; Self-optimization functions, protection, and healing and dealing with crucial network management challenges. These challenges are presently being addressed manually by special network operators. SELFNET will reduce remarkably the operational cost by improving user experience.
- Maximizing the future potentials and services of 5G advanced network infrastructures and its services based on cognitive management in NFV/SDN-enabled 5G networks using an End-to-End Cognitive Network Slicing and Slice Management Framework in Virtualized Multi-Domain, Multi-Tenant 5G Networks (SLICENET) project [37]. One of its use cases was a smart city in Romania, in the city of Alba Iulia. It was implemented for remote water metering intelligent public lighting systems.
- Finally, the Small cEllS coordinAtion for Multi-tenancy and the intention of Edge services (SESAME) [38] project is to develop a new multi-operator-enabled small cell that integrates a virtualized execution platform for deploying VNFs and supporting self-management capabilities.

The cited existing and on-going research projects are addressing the issues related to the MANO features of 5G networking technology. With the help of FC, a fully integrated and autonomous Fog nodes management system that goes beyond the state-of-the-art approaches may be achieved. It will combine data monitoring and analysis. It was noticed from these projects that different types of IoT approaches have emerged, and the willingness of industries to leverage or use their services is increasingly growing [21] alongside the management and decision-making operations for smart cities.

## 4. Computing Paradigms

Computation Technology has reached a new height with the advent of CC. Many service providers like IBM, Google, and Amazon are currently nurturing this computing paradigm to enable other cloud-based services and resolve numerous enterprise and educational issues concurrently. Cloud data centers are centralized and located far from the end users/ devices. one of the extensions of CC and FC are known as mobile EC (MEC) [7,38], which is edge-centric computing that is an evolution of cellular base stations. It combines the operations of cellular base stations and edge servers. With distant Cloud datacenters, MEC can be either online or offline. Moreover, it supports two or three-tier hierarchical application deployment of the network along with end mobile devices. Thus, targeting faster initiation of cellular services for the customers and improves network efficiency [38]. Recently, improvement has been made in MEC to support 5G communications. Moreover, it aims at flexible access to the radio network information for application development and content distribution. Another recent computation trend is Mobile Cloud Computing (MCC), and the user prefers to run necessary applications on their devices rather than traditional computers. The reason is that the recent proliferation of hand-held devices readily available today. However, most of the smart mobile devices are constrained with the subjected amount of energy storage and computational resource.

Executing intensive applications outside mobile devices is more feasible than locally executing applications devices, and remote execution of offloaded mobile applications is supported by the provision of computational resources in closed proximity of end users. Sensitive and real-time computation is leading to service quality degradation, round-trip delay, and network congestion. This issue was resolved with the help of the EC concept [38]. The basic concept of EC is to bring close data sources to the computation facilities, which is done by enabling data processing at the edge network as a localized computing paradigm [8]. Thus, a swift response to the request is needed for raw bulk data sent to core networks. However, it does not spontaneously give a response to other cloud-based services that are focused on the requirement of the end users [1]. In summary, MEC integrates Mobile and Cloud computing with wireless communication networks. It is performed to improve the Quality of Experience (QoE) and creates new business opportunities for both cloud service providers and network operators. MEC and FC can enable the edge network. Precisely, FC is a component of computational infrastructure that can be extended for both core and edge networks. It is evident from existing studies that FC supports IoT, Artificial Intelligence (AI), 5G, and many other programs that require high network bandwidth, advance security, constraint resources, and ultra-low latency. FC is the process of extending computing enterprise networks to the edge rather than working and hosting from a centralized cloud. It allows smooth computing operations, increased storage, and better network services between end users and computing data centers. Mostly, Fog computing is confused with edge computing. However, FC leverages EC but not the other way around as presumed by many people. FC gives way for orchestration, distribution, management, and services to secure resources across or between devices residing at the edge of a system-level architecture [7]. Contrasting to the concept of FC, EC places small servers or clouds at the edge by relying on separate nodes, where each node runs in a silo that requires data transported back through the cloud for peer-to-peer traffic [8]. As a result, the latency for service delivered to real-time IoT applications will be minimized to a great extent. FC can extend cloud-based services like IaaS, PaaS, SaaS to the edge of the network, unlike EC. thus, it is better to consider a good potential and well-structured computing technology for IoT to be compared as well as an enabler to 5G network technology compared to other computing paradigms. A SWOT analysis is explained in this study to have a summary of the future industrial requirement of the EC and FC.

Some challenges have been identified from the computing technologies, as summarized in Table 2. It is difficult to analyze and run data in the cloud in today’s digital world. Some critical issues such as latency, reliability of network bandwidth, and security, and some issues have been experienced in the edge with intelligent endpoints. The reason is that the reliability, storage capacity, and security still have critical challenges. However, FC is seen as a promising extension of the CC paradigm since it complements existing technologies. FC handle IoT related issues perfectly to enable the next generation of computing networks. Thus, it is evident that Fog computational nodes are heterogeneous and distributed, which must deal with different aspects of the constrained environment. Analyzing the features of Fog computing from structural, service-oriented, and security perspectives is pertinent. These exist many loopholes that need to be corrected for improved interoperability of the 5G networks with the concept of FC.

## 5. Overview of Fog Computing

The term ‘Fog computing’ is formed by ‘Salvatore J. Stolfo’ that was later picked up by ‘Cisco’ and is widely accepted by everyone. As described earlier, it is an extension of CC as well as services to edge network that allows other applications to run in proximity with the user’s device [39]. Due to this characteristic, service request latency is cut down, and QoS is improved, resulting in Quality of user experience. The help of fog computing bridges the continuum of cloud to things by communicating, controlling data from data sources. These will in-turn, enables faster processing of data as well as lowering network cost. Moreover, it supports many applications, especially time-critical (sub-millisecond reaction time) applications that need rapid latency. For the emerging Internet of Everything (IoE) applications that demands predictable latency, computing is a necessity for big analytics. Points of data collection in Fog computing are densely distributed geographically. Hence, a fourth axis geo-distribution is added to state-of-the-art Big Data dimensions (volume, variety, velocity, and veracity) [39].

Fog computing may not be as complicated as it sounds. At the network edge, software developers either write or port IoT applications on Fog nodes. The Fog nodes ingest data from the user or IoT devices from the network edge, which is the closest to the Fog nodes. Different types of data are directed to optimal areas where the analyses are needed. Time-sensitive data is analyzed on Fog nodes that are closest to data source [7]. Moreover, centralized aggregated node clusters are analyzed for data that can wait for seconds or minutes for the action to be taken. For data analysis and storage purposes, data with a lesser time sensitivity is sent to the cloud. A typical example is the data sent periodically from Fog nodes to the cloud for Big data analysis or historical storage purposes.

### 5.1. WM-Fog Overview

The challenges of Fog software architecture can be resolved using WM-FOG, which is a computing framework for the Fog environment. WM-FOG is designed to implement flexible software architecture. It can be incorporated using different user-specified policies or design choices.

**Figure 2:**
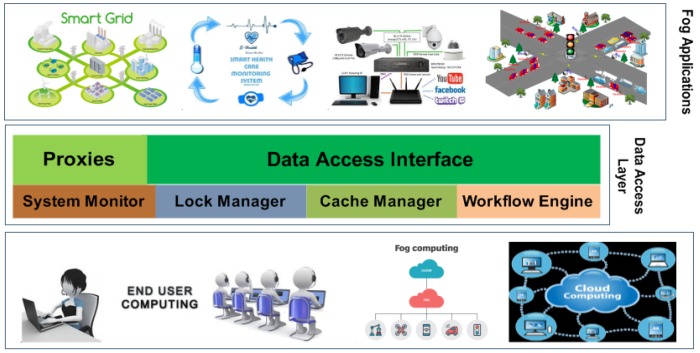
WM-FOG software stack.

More importantly, WM-FOG offers easy workflows that can deploy Fog-based systems. It can be implemented using properly scheduling workflows of the system entities such as Fog nodes, client devices, and back-end cloud servers [40]. WM-FOG leverages the FC paradigm to achieve a significant performance enhancement in providing better workflow as well as improving developers’ use of any underlying hardware resources. Workflow can be defined as; developer specifies its computations and data, and these data are known as ‘data item’, and computations are known as ‘computation transitions’ in WM-FOG. Every workflow contains one or more data items or zero or more transitions. Figure 2 illustrates the WM-FOG software stack, where the top layer is the application layer in which user applications reside. The next layer is the workflow layer, where workflow instances reside, such as the data access interface. Under the workflow layer, there is the system layer, where the system components reside. The bottom layer is known as an entity layer, where the system entities (client devices, fog nodes, and the cloud) reside.

### 5.2. Fog Physical Network Architecture

**Figure 3:**
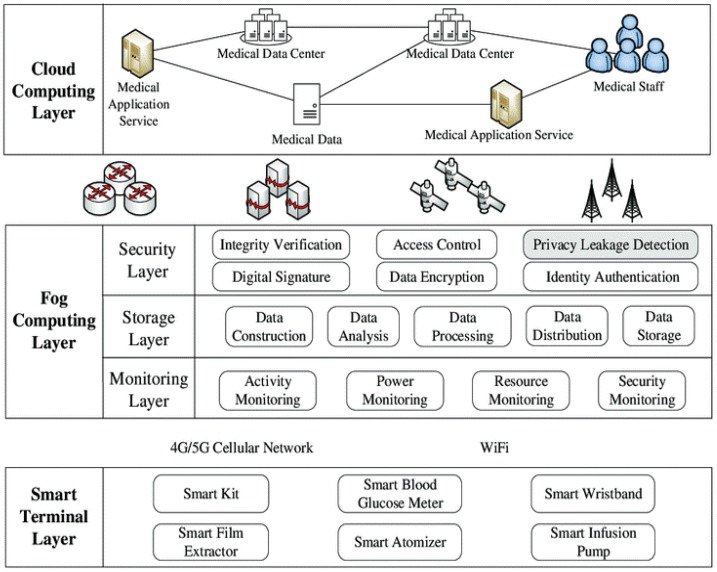
A three-layered Fog Computing network architecture with 5G network.

The state-of-the-art Heterogeneous Cloud Radio Access Networks (HCRAN) is extended by the Fog computing physical network architecture over 5G networks. It requires the processing of billions of end users’ devices to communicate data in HCRAN. This huge amount of data may interrupt the capacity front haul, which in-turn overburden the core network. It has an unfavorable influence on the QoS for the end-user devices. Thus, devices in every layer can provide and host computational storage by making it possible to create complicated offload processing policies. An illustration of the following Fog physical network architecture is shown in Figure 3.

#### 5.2.1. Application Architecture

- **Device Layer:** The description of how end-user devices are connected to the fog network in this layer. It includes IoT devices such as mobile devices (eMBB), sensors, gateways (mMTC), and actuators. Data transferred may perform data transfer with the network or directly with devices themselves (peer-to-peer). Moreover, this layer hosts computational tasks either by software running on the operating system or by embedded coding.
- **Fog Layer:** This layer consists of intermediate network devices located between the cloud layer and the end-devices (device layer). The Remote Radio Head is the initial point of offload in this layer and the small cell connected by fiber front haul to the core of networks, where incoming processing data reduces the burden on front haul. The macrocells from the point of offloading transmit processed data to the core networks via backhaul links. Intermediate devices and ethernet links are used for the realization of both the backhaul and front haul. It forms a potential area for computational processing and storage of data that can be offloaded.
- **Cloud Layer:** It is the apex in the architectural hierarchy, where the offload points are the cloud virtual machines. This layer can handle intensive computational processes with large storage that cannot be performed on edge computing due to the cloud layer’s high-end infrastructure and scalability. It contains a Based Band Unit (BBU) at the application layer processing data coming from the RRHs and small cells. The data passes through the first haul and route processed data to application servers [15,39]. FC architecture is known as decentralized, dynamic multi-layered, and resourceful, unlike another computing mode. Fog nodes act as a common network constituent linking the cloud, smart devices, and users with other Fog nodes.

#### 5.2.2. Emerging Technologies for Fog Networks

FC accommodates and allows three kinds of network connections, as given below; the wireless connections between edge devices and fog nodes, the wireless and wired connections among the fog nodes, and the wireless and wired connection between fog nodes and cloud data centers. These wireless communication technologies sustain fog applications. However, mobile fog computing supports the operation of Bluetooth, ZigBee, Wireless Local Area Network (WLAN), and 3G/4G. Fog computing architecture also supports many other communication technologies, and these technologies are further explained in the following subsections.

##### Network Function Virtualization (NFV)

NFV offers a novel approach towards creating, managing, and delivering network services. One of the prominent functions of NFV is that it detaches the activity of networks from the owned device by abstracting the device through virtualization techniques. It makes it possible for resource management of network services by delivering flexible services as well as fully ensuring the growth and improvement of new services [41]. NFV integrates varying virtualized parts or components like switches and gateways. These components can be virtualized and kept on fog nodes for easy resource management, storage, computation, communication of geographically dispersed fog networks as well as harmonizing other functions [42,43,44].

##### Software Defined Networking (SDN)

It is an emerging platform for networking that was designed to control programs via software applications intuitively. Its physical architecture allows it to delink the data component from its control component. It controls communication via centralized server route nodes [45]. Moreover, its physical structure allows flexibility, scalability, and capability of being programmed. SDN removes dependent embedded network devices such as switches and routers. It can also rid varying stemming from different network devices. Users are empowered to lay down their own rules and regulations of routing and transmitting networks, which provides flexible communication [46]. SDN plays a vital role in fog computing by competently managing diverse fog networks as well as offering solutions to reoccurring challenges [47]. These challenges include uneven connectivity and increased loss in packet data [48]. SDN can overcome the challenges in fog computing vehicle networks. It was proposed in the study [49], which suggests that SDN and fog computing supported a novel ad hoc network design known as FSDN for vehicles. The proposed study was aimed to improve the scalability, flexibility, uneven and poor connectivity-related network problems, and this is done by optimizing the use of resources and lessening latency.

##### Long-Reach Passive Optical Network (LRPON)

Long-reach Passive Optical Networks (LR-PON) technology is a better and improved version of the conventional Passive Optical Network (PON). The reason is that it saves costs to deploy fiber optics that are used in home buildings, offices, and roadways. LRPON network reaches stretched up to 100 km using several Optical amplifiers. It makes it easy for the consolidation of network processes over a wide area [50]. LRPON has a special use in fog computing, especially with regards to applications that are sensitive to latency and bandwidth, such as smart homes and smart industry services [43]. It is suggested that the enhancement of network design should assimilate fog computing and LRPON.

##### Content Distributed Network (CDN)

CDN is the bulwark that delivers content via proxy servers located at the edge of the Internet. It is an Internet-based cache network that takes the users and loads distance as factors of every node, as well as the connection status. The content that is in proximity to the user is then transmitted to the proxy server. Thus, this can curtail the information retrieved from either users or readers from faraway sites and improve the response speed [51]. CDN can provide many benefits when synched with fog computing attributes, these benefits like the reduction of costs and expenses, reduce the usage of bandwidth, improve the availability of more content, and it can reduce the congestion of the network. The incorporation of FC and CDN, especially when aligned with context-aware technology, can deliver outstanding services to end users [52].

##### Naming Identification Resolution Fog computing

It is known as a resource-studded center that accommodates a large number of applications, gadgets, and devices that operate to offer different services. Since a computer network has a Domain Name System (DNS), fog computing must also have a naming, identification, and resolution. It is expected to meet the requirement associated with controlling objects, data communication, the discovery of services, and verification of identity. An established competent naming mechanism like DNS and Uniform Resource Identifier (URI) are leveraged extensively in networks to collaborate among diversified applications, devices, and gadgets. However, the location of many devices, applications, and gadgets at the edge restricts their resources and render them decidedly mobile. It allows the mechanisms inflexible in several scenarios, especially for the sync with vibrant fog computing platforms. Additionally, the famous IP-based naming system could not be leveraged in this case due to the high cost. Consequently, several novel identifications or naming methods have been proposed to be coordinated with the functionalities of fog computing. These methods are known as Named Data Networking (NDN) [53] and MobilityFirst [54]. NDN is a growing IP design that considers “the contents (What) rather than the addresses (Where).” NDN can provide signatures to humans, computers, books, and sensors. Its packets are tier-layered data summons names rather than locations, destinations, and addresses. It is heavily focused on enhancing the competence, stability, safety, and scalability of prevailing computer networking paradigms. These features made it appropriate for edge and FC. The MobilityFirst aims to resolve the problems related to wireless mobility and access. It is aimed to fulfill the need for present mobile Internet requirements protocols. In contrast to the current systems, MobilityfIrst detaches names from addresses on the network by employing both Global Names Resolution Services (GNRS) and Global Unique Identification (GUID) to affix names and addresses together. Though, API services concentrate more on source network objects or names of destination, rather than the address network. MobilityFirst leverages the embedded name/address routing approach to be scalable. This approach delivers excellent results for devices that use in fog computing technology. The identification of devices, applications, and gadgets; this approach is categorized into three segments named Physical objects identification, Communication Identification, and Application Identification. These categories are further defined in the following sections.

- *The Physical object Identification:* It is also known as a non-ID verification code identifier that is chiefly concerned with the identification of application devices and gadgets. This kind of identification employs identifiers as ID code and natural property [55]. The natural property identifiers use biometrics, behavior attributes, temporal, spatial, and other information attributes as identifiers [56]. The ID code identifier, on the other hand, comprises of numbers and alphabets with some rules attached to them such as ubiquitous ID (uID) [57], European Article Number (EAN) and Electronic Product Code (EPC) [58].
- *Communication identification:* It is employed by verifying the identity of the application, devices, gadgets, and network nodes that can deal with communication such as E.164 number, Mac address, IP address, and others [59].
- *Application identification:* It is concerned with identifying several applications such as Domain Names and Uniform Resource Locator (URL) in fog computing platforms. The Object Name Services is known as the most popular identifier that used resolution services, and it is part of the EPC global network [60]. Object Name Services supports mobility attributes in fog computing. A study made advancement in fog computing by entrenching a framework to identify and resolve user’s faces. This study enhances the efficiency of processing and preserves bandwidth network services. The method was also able to double up reference for non-ID verification [61].

##### Storage Technologies

The functionality of pre-cache technology plays a vital role in fog computing. It fulfills the following requirements and produces low-latency responses. Fog nodes initially anticipate the demand of the users then intuitively select the best content to deliver in geographically dispersed nodes. However, delay in downloading contents from remote sites or data centers can be curtailed substantially by enabling fog applications to exploit storage resources to users’ excellent services [48] fully. A study has offered a dedicated caching mechanism that would supply information before users for demanding for it [62]. Therefore, the pre-cache mechanism can be incorporated in FC. It can be used in applications use cases like smart vehicle and traffic applications. These systems can proactively anticipate the demands of drivers and stash their devices in edge base stations. It will significantly reduce traffic demands, especially at peak moments. Edge devices have constricted storage capacity. Storage extension in fog is proven to be beneficial to improve the strength of fog computing technology. Another study proposed a novel concept of employing personal storage in mobile gadgets, as a method to extend the storage as well as to secure device component [42].

##### Security and Privacy Protection

Devices that have proximity with end users and employ fog nodes are sometimes carried to an unsecured location. These devices may be vulnerable to unwanted attacks such as the man-in-the-middle attack [63]. It means that fog nodes devices could be switched to fake fog nodes virtually. This challenge could be resolved through encryption and decryption approaches. Another pertinent challenge is data confidentiality and integrity. The dispersed devices mostly cause this at the edge, creating a huge volume of data that must be transmitted and stored in fog nodes for computational task and storage. Nevertheless, fog nodes interact with gadgets and data pools in cloud computing. These complex processes make data vulnerable to hijacking. There is a proffered solution to solve this problem, known as light-weight encryption algorithms and masking techniques [64]. In fog computing, there are problematic areas where collaboration takes place, which may lead to safety and privacy issues. These areas include resource access control, quality of service and security, secured collaboration, and distributed decision enforcement as well as information sharing policy [64]. A study put forth a policy based on resource management and access control system to ensure that users have secured interoperability and partnership with diversified resources [45].

##### Management of Resources

Resource management is paramount in FC for leveraging and managing fog services and resources. It has been noted that there is normally restricted energy remaining in fog nodes and devices at the edge. Therefore, the management and allocation of resources have a significant influence on the durability and performance of fog network. However, to improve the processes of low-latency and mobility in fog computing, the methods of resource management need to be concentrated. It is especially required when factors such as fog nodes, migration, placement, device strengthening at edge, application module needed to be well studied. Moreover, these factors have their adverse effects during the processing of latency and when the decision is meant to be made. Some of the solutions are suggested to resolve this issue as; the resource virtualization of fog nodes may improve resource management competency. Another approach involves enabling context-awareness technology for services and management of resources in fog computing. The management of energy and resources within the domain of context-awareness technology may ensure improved usage of resources and energy conservation [65]. It is important to note that resource discovering and sharing in fog computing are paramount for application performance enhancement. It can alternate flooding and centralized modes naturally by conserving energy from diverse networks. A robust mobile cloud computing resource discovery approach was proposed to transform between flooding and centralized strategies in saving energy from diversified networks automatically [66].

## 6. Use Cases of Fog Computing

As the marketplace continues to deliberate how best to incorporate IoT devices with 5G networks. One of the most daunting tasks or issues is how fog computing can be leveraged to play a vital role in this process. Before understanding these roles, it is important to know the taxonomy of how fog computing enables IoT. Therefore, studying these taxonomies will better the understanding of fog architecture to refine requirements and the demonstration of fog values to customers. The concept of applying FC in IoT applications is imprecise, as described in an online web page published by Cisco blogs [41]. This webpage describes the applicability of Fog use cases in IoT with titled “How we use Fog Computing: Vertical Markets, Use cases and application” published on the 11 May, 2018 by ‘Helder Antunes.’ It is better to broaden this concept into different taxonomies, namely verticals, applications, and use cases for precise and better understanding of how fog computing can be applied to IoT [41].

Verticals are entire domains of the Internet of Things marketplaces such as Network domains, Industries, or special classes of users [37]. Verticals have appropriate environmental regulatory policies, procedures, and protocols that are widely used. Some of the verticals pertinent to the domains of IoT and Fog computing include; Logistics, Smart building, Utilities/ Energy, Health care, Data centers, Agriculture, Government [7], Military, Residential, Ubiquitous Computing, Manufacturing, Oil, and Gas. The next phase of taxonomy is the Use cases. This phase is a subdivision of the verticals where every vertical is a segment of IoT that can be served as a single IoT platform. For instance, in the transportation vertical, there may be related use cases on autonomous vehicles [19], smart highways, smart railways, and many more. The interaction of the IoT network between use cases may be more within a single vertical than there is between verticals. An Organization that develops fog software or hardware for a specific use case within a vertical can often employ that expertise to perform another use case in similar vertical effectively. The third phase is the Application phase, which represents special hardware/software solutions that provides IoT capabilities for consumers/clients/customers. Using the transportation vertical as an example. The smart railway use case can employ applications like the cargo tracking signaling, Positive Train Control (PTC) safety system, crew communications, and others. The railway services may want to install some of the selected applications on its fog network, perhaps acquiring services from multiple suppliers. Applications may be an appropriate component to provide models for commercial use to establish an effective marketplace for fog. These include Google play store or iTunes App store but with a focus on IoT and fog apps. As the application marketplace develops, special developers with domain experience would begin to drive the exponential growth in IoT and fog software [41].

**Figure 4:**
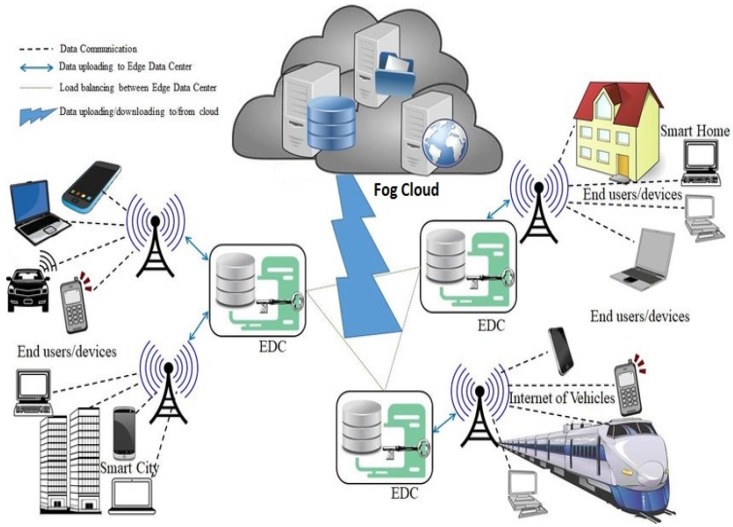
Applications of Fog Computing.

- **Remote monitoring for Oil and Gas operations:** Monitoring of Oil and Gas operations using state-of-the-art centralized data analytical approach to predict causes of downtime in operation or failure based on available trained datasets may not be the best approach to prevent disasters in oil and gas operations. However, setting up near-instant rules at an operations site that can analyze data and see signs of disaster before it happens to take adequate measures for prevention is needed. In this case, Fog computing fits-in since it is built on the edge with closer proximity to nodes where information is shared. It sees the sign of events, either normal or abnormal (disaster), and takes appropriate measures to prevent catastrophe before it starts.
- **Retail customer behavior analyses:** FC can be used for near-instant analytical retailing to reduce cart abandonment as well as improve customer engagement. Sales data such as images, coupons, and so on provide an insight into customer behavior. This information or data can help retailers improve target merchandise as well as sales and promotions.
- **Self-Driving Cars:** With the advent of Advanced Driver Assistance Systems (ADAS), the next-generation self-driving cars or vehicles will become safer as ADAS increasingly grows to understand as well as react to environmental conditions. The real breakthrough in self-driving cars is the democratization of ADAS technology. In other words, this means the availability of ADAS technology to everyone (commercial vehicle, premium vehicles, first-time and senior drivers). Cars must compete with the development and deployment of next-generation computing technologies. Fog computing allows the car to leverage a common set of systems that can deliver a better user experience. Other typical use cases of Fog computing can be seen in Figure 4.

Figure 4 illustrates the use of fog in IoT, putting all these concepts together. It is assumed that a great organization of IoT networks will employ fog computing. The OpenFog Consortium is in the process of building fog nodes and networks that would be versatile enough to provide verticals from the listed use cases as well as a huge spectrum of applications serving these use cases [8,42,43].

## 7. Convergence of 5G and Fog Computing

A major way in which 5G networks can enable fog computing is the introduction of advanced network technologies for increased bandwidth spectrum, advanced modulation schemes with algorithms, and enhanced hardware technology to handle the proposed technological requirements. Advanced MIMO antenna modules, routers, and C-RAN units must be deployed for smart applications. Moreover, Wi-fi offloading is an essential technique to offload data traffic from small cell base stations. These technologies are further described in the following sections.

**Small cell or Low Powered Radio Access Nodes (RAN):** It ranges between 10 m and 2 km. It is installed in a densely populated urban region, which may not be able to support macrocells. However, its functions exist either in unlicensed or licensed spectrums. A suited Fog RAN semi-hybrid distributed resource allocation algorithm was proposed by [5]. It is a periodical cloud BBU that is centralized for carrying out user pre-scheduling as well as a distributed local beam-forming frame at each Fog AP. However, the joint algorithm that is centralized to resolve beam-forming and user clustering in CRANs can only leverage imperfect CSIs due to the inevitable transport delays on front-haul links. Hence, the proposed algorithm makes use of perfect CSIs at Fog Aps despite local optimization for beam-forming and large-scale cloud processing to improve imperfect pre-scheduling of CSIs. Simulated results of the proposed method in terms of delay and throughput showed a practical, realistic level of imperfect CSI. Delay improvement suggested that small packets are well suited for future IoT applications [32]. Several users are directly served by the broadcast downlink Macro-cell-Based Station (MBS), while others served directly with the Small Cell-Based Stations (SBSs) side channel that is also known as “side-link.” The basic trade-off for the Fog Radio Access Network (F-RAN) model includes:

- Size of the cache memory at the SBS
- Load of MBS downlink served directly to user devices and SBS
- A study on the aggregate load of side-link under a standard demand of worst-case scenario.

Previous studies recovered special cases of this network model, and by-product results of independent interest are given. From the FC perspective, both small and macro-based stationed cells can be employed to attain ultra-low latency needed by the 5G networks. These network nodes could provide storage and computation power too. A fog computing-based radio access network (F-RAN) could provide 5G networks with high spectral and energy efficiency as well, drawing from capabilities in edge devices like local radio signal processing and distributed storage [1]. It can provide a more effective data rate and low-latency network coverage for users in every unit. However, to increase data rate, Multiple Input, Multiple Out (MIMO) concept can be enhanced as well. It is discussed briefly in the next section.

**Multiple Input, Multiple Out (MIMO):** MIMO concept is employed to increase the data rate to transmit and receive signals with the help of additional antennas. A huge number of miniature-sized antenna arrays are used in 5G technology. Moreover, multiple antennas integrated with mobile devices will be used to send and receive data signals. A novel Fog Computing technique in the uplink of the front-haul constrained Distributed Massive MIMO system (DM-MIMO), and the corresponding power control algorithm [33]. By performing decentralized signal detection, followed by user-based compression at distributed enhanced RRHs, the amounts of overheads, the number of quantizers, and the overall optimization complexity can be greatly decreased.

**Offloading:** Data will exponentially continue to increase with the advent of 5G networks. However, Internet Service Providers (ISP) are seeking measures to prevent congestion on Internet usage. Offloading data traffic free up network capacity as well as providing high QoS. In this section, some reviews are made of recently proposed offloading techniques in fog computing based on different modalities. In [34], the authors proposed a scheme that is based on transmission time and energy consumption. This scheme was applied to a mobile device communicating with fog. This fog can forward the task to the cloud or execute it on its own. If executed on its own (within the fog), it experiences transmission delay of inputted data, and execution time is dependent on the processing power of the fog. If the processing task requires offloading to the cloud, delay remains the same. However, the transmission delay will include a communication from fog to cloud. Moreover, the energy consumption by fog in delegating executed tasks to cloud and energy consumption by the cloud to execute the task is used up. In a fog-only scenario, total energy consumption is based on idle energy consumption of user devices that is necessary to transfer input bits to the fog. In an event where the cloud is also involved, the additional energy consumption would be that of fog transmitting the tasks to the cloud and the cloud then executing them.

After reviewing the importance of the previous and existing state-of-the-art fog computing and networking technologies, it is possible to point out a set of technologies that may be the fundamental enablers to the advancement of Fog computing for the 5G networks. In the case of cloud computing, it is known that virtualization has been used to power cloud technology. The next question is what major technology powers for fog computing. Since fog computing is starting to re-sharp the future of computing network landscape.

### 7.1. The Needs for Fog and 5G Network Technologies

Several modules are needed to be added to FC and 5G cellular network technologies to provide better data transmission services. These modules are thoroughly explained below:

#### 7.1.1. The Physical Object Identification

It is also known as a non-ID verification code identifier that is chiefly concerned with the identification of application devices and gadgets. This kind of identification employs identifiers as ID code and natural property [55]. The natural property identifiers use biometrics, behavior attributes, temporal, spatial, and other information attributes as identifiers [56]. The ID code identifier, on the other hand, comprises of numbers and alphabets with some rules attached to them such as ubiquitous ID (uID) [57], European Article Number (EAN) and Electronic Product Code (EPC) [58].

#### 7.1.2. Communication Identification

It is employed by verifying the identity of the application, devices, gadgets, and network nodes that can deal with communication such as E.164 number, Mac address, IP address, and others [59].

#### 7.1.3. Application Identification

It is concerned with identifying several applications such as Domain Names and Uniform Resource Locator (URL) in fog computing platforms. The Object Name Services is known as the most popular identifier that used resolution services, and it is part of the EPC global network [60]. Object Name Services supports mobility attributes in fog computing. A study made advancement in fog computing by entrenching a framework to identify and resolve user’s faces. This study enhances the efficiency of processing and preserves bandwidth network services. The method was also able to double up reference for non-ID verification [61].

#### 7.1.4. Storage Modulation

The functionality of pre-cache technology plays a vital role in fog computing. It fulfills the following requirements and produces low-latency responses. Fog nodes initially anticipate the demand of the users then intuitively select the best content to deliver in geographically dispersed nodes. However, delay in downloading contents from remote sites or data centers can be curtailed substantially by enabling fog applications to exploit storage resources to users’ excellent services [48] fully. A study has offered a dedicated caching mechanism that would supply information before users for demanding for it [62]. Therefore, the pre-cache mechanism can be incorporated into fog computing. It can be used in applications use cases like smart vehicle and traffic applications. These systems can proactively anticipate the demands of drivers and stash their devices in edge base stations. It significantly reduces traffic demands, especially at peak moments. Edge devices have constricted storage capacity. Therefore, the storage extension in the fog is proven to be beneficial to improve the strength of fog computing technology. Another study proposed a novel concept of employing personal storage in mobile gadgets, as a method to extend the storage as well as to secure device component [42].

#### 7.1.5. Security and Privacy Protection

Devices that have proximity with end users and employ fog nodes are sometimes carried to an unsecured location. These devices may be vulnerable to unwanted attacks such as the man-in-the-middle attack [63]. It means that fog nodes devices could be switched to fake fog nodes virtually. This challenge could be resolved through encryption and decryption approaches. Another pertinent challenge is data confidentiality and integrity. The dispersed devices mostly cause this at the edge, creating a huge volume of data that must be transmitted and stored in fog nodes for computational task and storage. Nevertheless, fog nodes interact with gadgets and data pools in cloud computing. These complex processes make data vulnerable to hijacking. There is a proffered solution to solve this problem, known as light-weight encryption algorithms and masking techniques [64]. In fog computing, there are problematic areas where collaboration takes place, which may lead to safety and privacy issues. These areas include resource access control, quality of service and security, secured collaboration, and distributed decision enforcement as well as information sharing policy [64]. A study to provide the solution for the challenges mentioned above puts forth a policy based on resource management and access control system to ensure that users have secured interoperability and partnership with diversified resources [45].

#### 7.1.6. Management of Resources

Resource management is paramount in fog computing for leveraging and managing fog services and resources. It has been noted that there is normally restricted energy remaining in fog nodes and devices at the edge. Therefore, the management and allocation of resources has a significant influence on the durability and performance of fog network. However, to improve the processes of low-latency and mobility in fog computing, the methods of resource management need to be concentrated. It is especially required, when factors such as fog nodes, migration, placement, device strengthening at edge, application module needed to be well studied. Moreover, these factors have their adverse effects during processing of latency and when decision is meant to be made. To resolve this issue, some solutions are suggested as; the resource virtualization of fog nodes may improve resource management competency. Another approach involves enabling context-awareness technology for services and management of resources in fog computing. The management of energy and resources within the domain of context-awareness technology may ensure improved usage of resources and energy conservation [65]. It is important to note that resource discovering and sharing in fog computing is paramount for application performance enhancement. It can alternate flooding and centralized modes naturally by conserving energy from diverse networks. A robust mobile cloud computing resource discovery approach was proposed to transform between flooding and centralized strategies in saving energy from diversified networks automatically [66].

## 8. Future Directions of Fog Computing to Enable 5G

A sustainable technology optimizes the environmental influence and economic value of fog computing to a reasonable extent. However, the sustainability of the overall FC architecture is subject to varying challenges such as reusability of services, efficient energy resource management, and the assurance of the quality of services. On the other hand, the consistency of fog nodes can be termed as reliability. It could also mean fault tolerance, high-performance services, and secured interactions [39]. In past studies, a small discussion on the reliability and sustainability fog computing has been provided as follows:

- It is highly recommended that more research should be performed to improve the performance of fog computing besides the state-of-the-art networking activities such as packet forwarding, routing, and switching. In some cases, fog nodes have special networking components by performing more like a computational component instead of performing traditional networking activities. In other scenarios, the networking activities or capabilities of fog nodes become more prominent than its networking capabilities [22].
- Interoperability of fog nodes with 5G architecture can be self-customized according to the needs of the user. From previous studies, many special fog nodes have been proposed. Still, there is a need for real interoperable fog nodes architecture to be investigated, developed, and deployed for real-world testing. The overall data produced by IoT devices is increasingly becoming big data. According to Big Data, challenges can be divided into two main folds, which may lead to the efficient storage of data activities and semantics extracted from the stored data. The semantic data extracted can be used for meaningful and useful information from the massive volume of information produced by [35].
- It is known that fog nodes are distributed across edges, but not all the fog nodes are resourceful. Large-scale deployment on single fog nodes is not often feasible. Therefore, an effective solution is needed, like the modular development of large-scale applications, as well as their distributed deployment over constrained fog nodes is an effective solution along with 5G approaches. Previous studies on fog computing proposed different platforms for developing and deploying distributed applications. Thus, the challenges regarding the deployment of distributed applications like latency management, assurance of QoS, dataflow management, and affinity of real-time based on edge-centric applications are yet to be properly addressed [67].
- For future studies, the development of an efficient simulator for the fog-5G network is a basic need to accomplish. FC is basically designed to extend cloud services to the proximity of IoT devices. An extensive modification and improvement of existing programming languages and standards are highly required to enable cloud services in Fog, as the architecture of Fog is quite different from the cloud, [68]. Moreover, the development and management of seamlessly flexible large number of connections in fog computing for efficient networking protocols and user interfaces are necessary. Many fog-based types of research consider works on contextual information to estimate the resources and other important aspects of Fog that are unexplored.
- A hierarchical 5G VANET architecture can be developed by emerging the concept of FC approaches. It can be developed at the edge of the network. Therefore, the distributed architecture will support the 5G network technology in terms of location awareness, real-time services, and delay-sensitive that will be suitable for future 5G networks. Moreover, it will reduce the data transmission latency, overhead, operating cost, and it will improve the throughput of the emerging network.
- FC and 5G network technologies soon will face difficulties in providing efficient data, dissemination services, and applications. The reason is that the traditional service composition technologies that were provided to the cloud services will not be acceptable in the future regarding effective Fog service and forthcoming large scale. Even these approaches were not coping efficiently with high mobility, reduced delay, real-time execution, and high scalability. Therefore, there is a need to develop an emerging (5G and FC) network architecture to deal with these arising issues in the future. The emerging network can be deployed among the client devices with high mobility. Thus, the emerging network approach will enhance the quality of services.
- The FC technology can emerge with a 5G network in terms of making a cellular base station as a Fog server with its capabilities of storage and computing facilities. Therefore, the whole Fog network will provide adequate coverage and enhanced services to cellular users [69].
- FC wireless approach can be applied as a centralized resource pooling to improve operational efficiency and services. Fog-based wireless networking approaches can reduce data transmission delays by the implementation of the processing system at the edge of a network. Therefore, the Fog with 5G network will enhance the data dissemination services in the radio access network, and it can also support the diverse number of applications [70].

## 9. Conclusions

Fog computing (FC) is one of the technologies which has gained rapid growth in a brief period. FC enables the effective processing of data retrieved from smart devices. It brings computing paradigm from the cloud to the edge of the network with computing capabilities. However, massive data generation from numerous sensors and actuators in the Internet of Things (IoT) has first networks to a bottleneck. Thus, the need for an improved computing server architecture to handle the continuous growing demands of smart devices is pertinent. A better model is required to enable smart devices to be able to use the on-going 5th Generation (5G) networks. 5G network is envisaged to enable rich connections as well as smarter data processing on user devices. It is the key to an autonomous world of computing, where user devices can operate independently with direct human control. Therefore, in this study, some cases of fog computing on 5G networks are discussed. Currently, using FC technology in 5G systems is has much vision, and the same goes for the 5G network itself. Therefore, to actualize this vision, a few of the research areas needs to address. The concept of Network Function Virtualization (NFC) in heterogeneous networks using FC as an enabler to 5G is not clear in the present times since there is a need for improvement in implementing NFV for mobile communication networks as explored in earlier studies.

Moreover, a large amount of energy is required for the processing of more substantial applications. Minimizing energy consumption is a key for commercializing FC technology in 5G systems. Another challenge is the pricing policies because different parties require different prices of resources for the use of FC services. Thus, finely granulated accounting management and price policy are needed in resource management of the FC and 5G ecosystem. Finally, the issue of visibility of data to the third party by 5G networks makes privacy and security a pertinent point in FC architectures. These issues and challenges can be addressed using FC in 5G networks. It is evident that both technologies, i.e., is FC and 5G, are compatible with each other. However, combining both concepts is envisaged to be the future of Internet services. The present study aims to find a better solution and a more sustainable future for humanity.

## Figures and Tables

**Table 1:** A few of the related studies in Fog Computing as an enabler to 5G networks.

Reference	Description of Related Existing Studies with Contributions
Barakabitze et al. [11]	A comprehensive survey of 5G slicing with SDN, NFV, FFC, and ENC networks is presented. In this survey, the use cases of 5G related to network softwarization and slicing are defined in which Virtual Machine FC, SDN, network Hypervisors, NFV, and MEC are included. Therefore, a comparison of 5G approaches to adopt modern technologies related to industry and academia are presented.
Aggarwal et al. [12]	A survey of FC and 5G related to Tactile Internet is presented. The article provides knowledge about the issues and challenges related to Tactile such as data transmission delay, energy, and security. In addition, this survey focuses on communication infrastructure, healthcare, resource management fog orchestration, privacy, and security related to Tactile Internet applications. Moreover, it brings an overview related to scalability, quality of service, energy efficiency, interoperability, and mobility under the umbrella of Tactile Internet applications.
Akpakwu et al. [1]	The current state-of-the-art features of IoT were reviewed in this paper. Basic infrastructures like the cellular-based LPWA eMTC, NB-IoT, EC-GSM-IoT, and non-cellular LPWA technologies like SigFox, LoRa, and Ingenu-RPMA with focus on 5G networks as next-generation network to enable new service requirement.
Asrar et al. [15]	An overview of the identification of important research issues in different fog computing architectures were studied in this research. Fog computing is presaged as the next computing paradigm since it applies to a wide range of application networks. Literature was reviewed in this study to identify different roles played by fog computing in describing 5G network slicing covering three main application domains, known as, uRLLC, eMBB, and mMTC. Also, it discusses other research challenges, directions, and the purposes of FC with a focus on its role in emerging technologies.
Santos et al. [8]	The main contribution of this paper is the full integration and autonomous management system of fog node for data analysis and decision-making purposes. Alongside this, the paper experimented on a new application layer P2P fog protocol based on the OSPF routing protocol. It aims at communicating information between cloud layers and fog nodes. The study framework sends timely alerts to Internet of Things sensors whenever there is a detection of deviant samples.
Amendola et al. [5]	A novel approach was presented in this paper to manage bandwidth in the live migration of wireless virtual machines. Results indicate a significant improvement with regards to the used approach in the most useful implementation of the architecture for virtual machines.
Yousaf et al. [16]	To realize the vision of 5G by 2020, many research challenges need to be addressed like; How to manage software agility, especially in the perspective of network functions as micro-functions. How network functions are distributed into many executable platforms and when programmable hardware (smart NICs and switches) becomes more ubiquitous. How to ensure the interoperability between different vendors, especially in this cloud-native environment of massively decomposed network functions. How to map and translate business requirements of clients efficiently over the infrastructure of service providers. How to ensure that Quality of Service and Quality of Experience requirements with SLAs can be achieved in a cloud-native environment. How to manage and assign resources efficiently for the existing multitude slices in a similar administrative domain, or that are traverse over different administrative domains. To what extent can the management of network/system services be automated to reduce the need for manual tasks and intervention

**Table 2:** A brief SWOT analysis of Cloud, Edge and Fog computing.

Architectures	Strength	Weakness	Opportunities	Threats
Mobile Edge	It enables the convergence for both computational processes and connectivity of networks. It also enhances the service performance for application developers.	It is inefficient for optimizing resource use.	It is an advanced or novel revenue Stream for Service Providers. Fast and cost-effective service development.	Transparency in user migration applications may not be well secured.
Mobile Cloud Computing	It reduces cost with a scalable working environment. It provides a global approach with a secured data back-up and recovery mechanism.	There are issues related to contracts or service agreement of Internet service providers and challenges in data migration. Complexities in integrating with the existing architecture.	ERP-SaaS Mobile automated data gathering Mobility Security Improvement.	There are cases of data loss and privacy breaches. Also, there are cases of dissatisfaction with offerings/ performance/ pricing from vendors Legal and regulatory.
Fog Computing	It consists of a vast distribution of controlled systems with a few actuators and sensors working together to improve the quality of user experience. It complements both edge and cloud computing technologies. Data collection points are widely distributed (Geo-distributed applications).	It is prone to DOS Attack since resources constrain fog nodes. A huge sum of concurrent requests is daunting to manage.	Servers are built on the edge of networks to prevent. Security is user-defined or user-based. Location awareness.	Due to heavy powered data processing, there is insufficient energy to power Fog nodes. Privacy Authentication. Dissatisfaction pricing from vendors

## Abbreviations

The following abbreviations are used in this manuscript:

FC	Fog Computing
5G	Fifth Generation
IoT	Internet of Thing
3GPP	Generation Partnership Project
eMBB	Enhanced Mobile Broadband
URLLC	Ultra-Reliable Low-Latency Communications
uMTC	ultra-Machine Type Communications
mMTC	massive Machine Type Communications
LPWA	Low Power Wide Area
URLL	Ultra-Reliable Low Latency
SDN	Software Defined Networking
5G-PPP	5G infrastructure Public–Private Partnership
NDN	Named Data Networking
API	Application Programing Interface
MBS	Macro-cell-Based Station
EAN	European Article Number
EPC	Electronic Product Code
